# Supplementary material for: Role of long intergenic non-protein coding RNA 01857 in hepatocellular carcinoma malignancy via the regulation of the microRNA-197-3p/anterior GRadient 2 axis
Source: PLoS One. 2021 Nov 18;16(11):e0258312. doi: 10.1371/journal.pone.0258312 (PMC8601473; doi:10.1371/journal.pone.0258312)

Full unedited image for Fig.6A

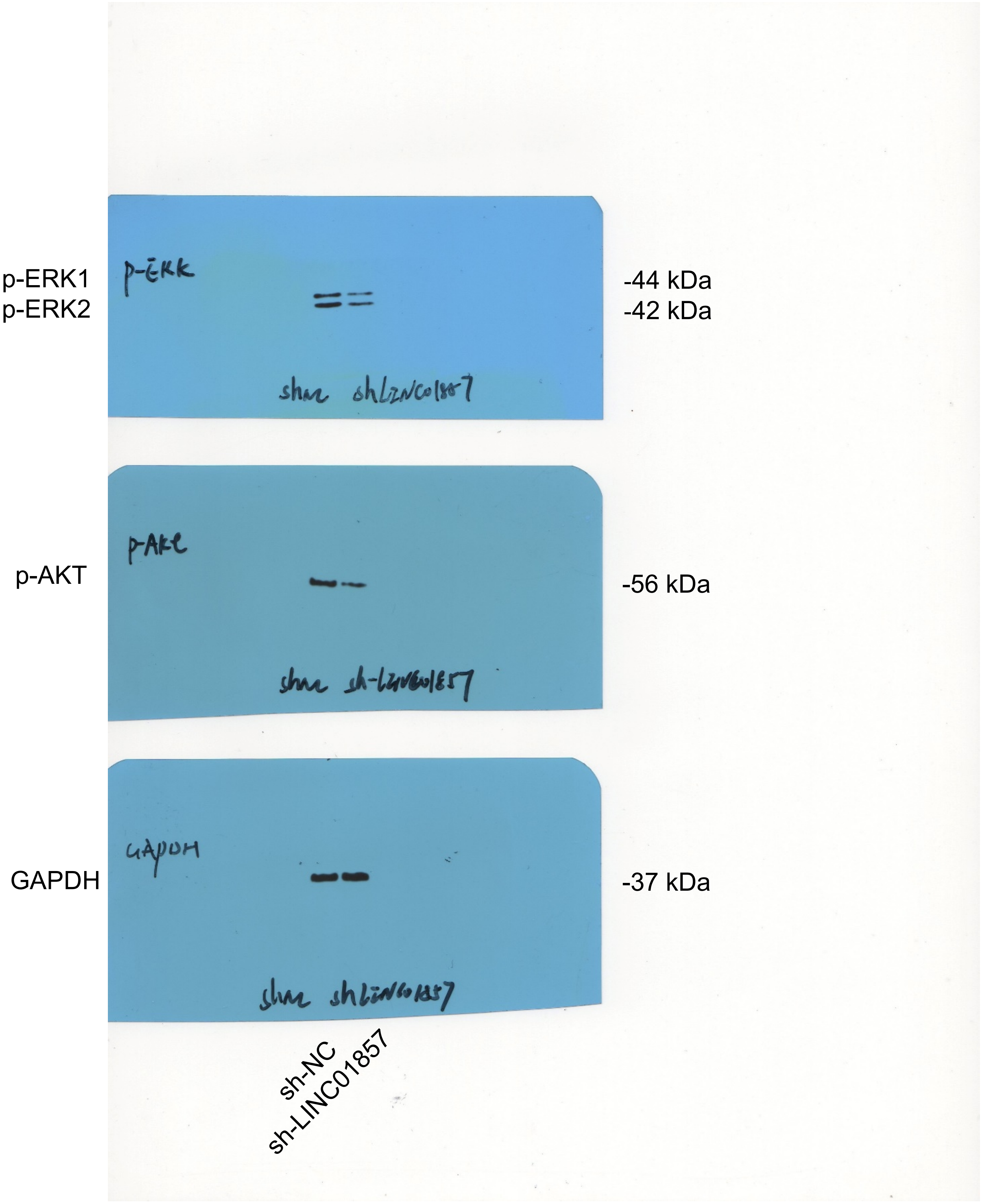


Hep3B cells

Full unedited image for Fig.6A

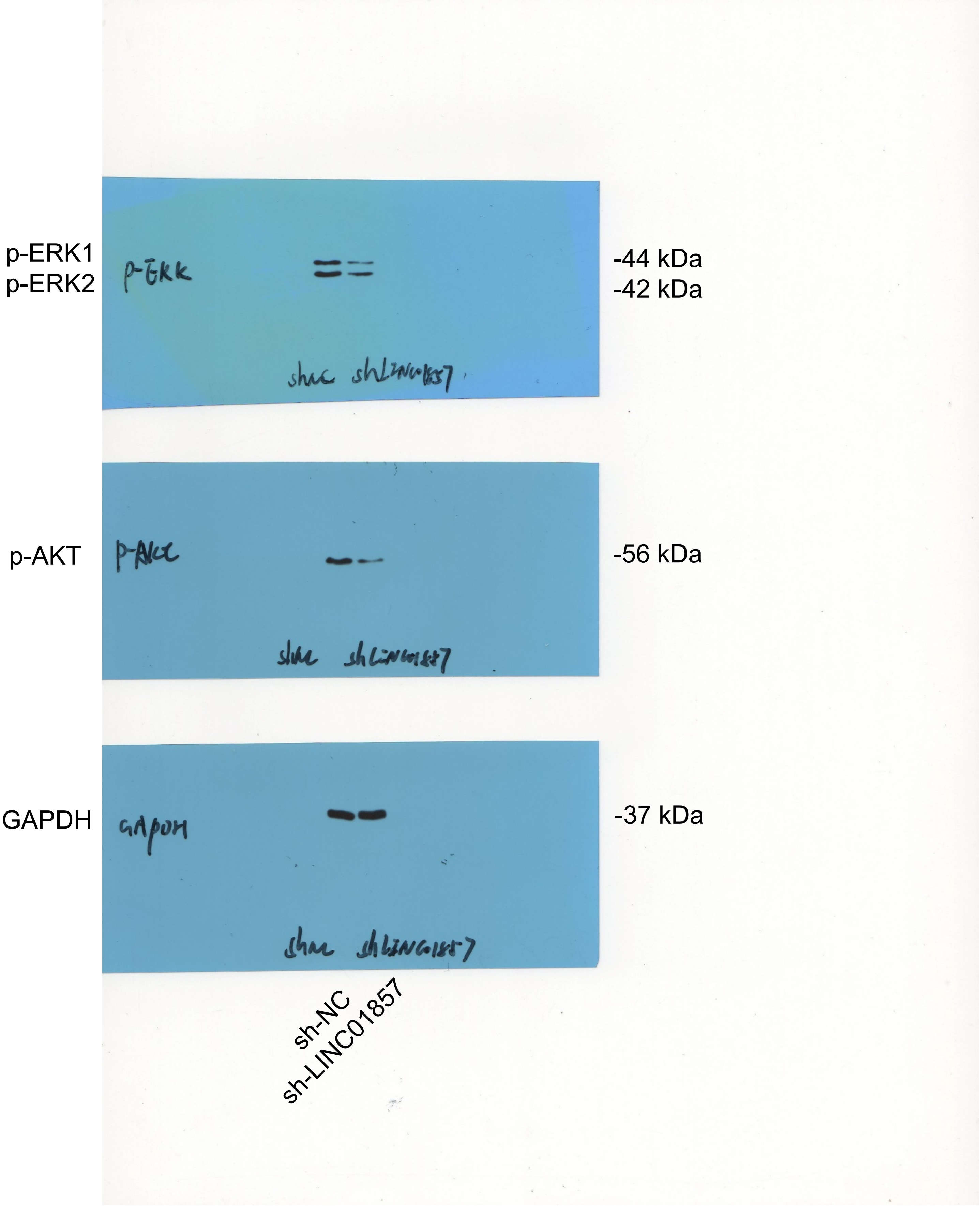


HepG2 cells

Full unedited image for Fig.6B

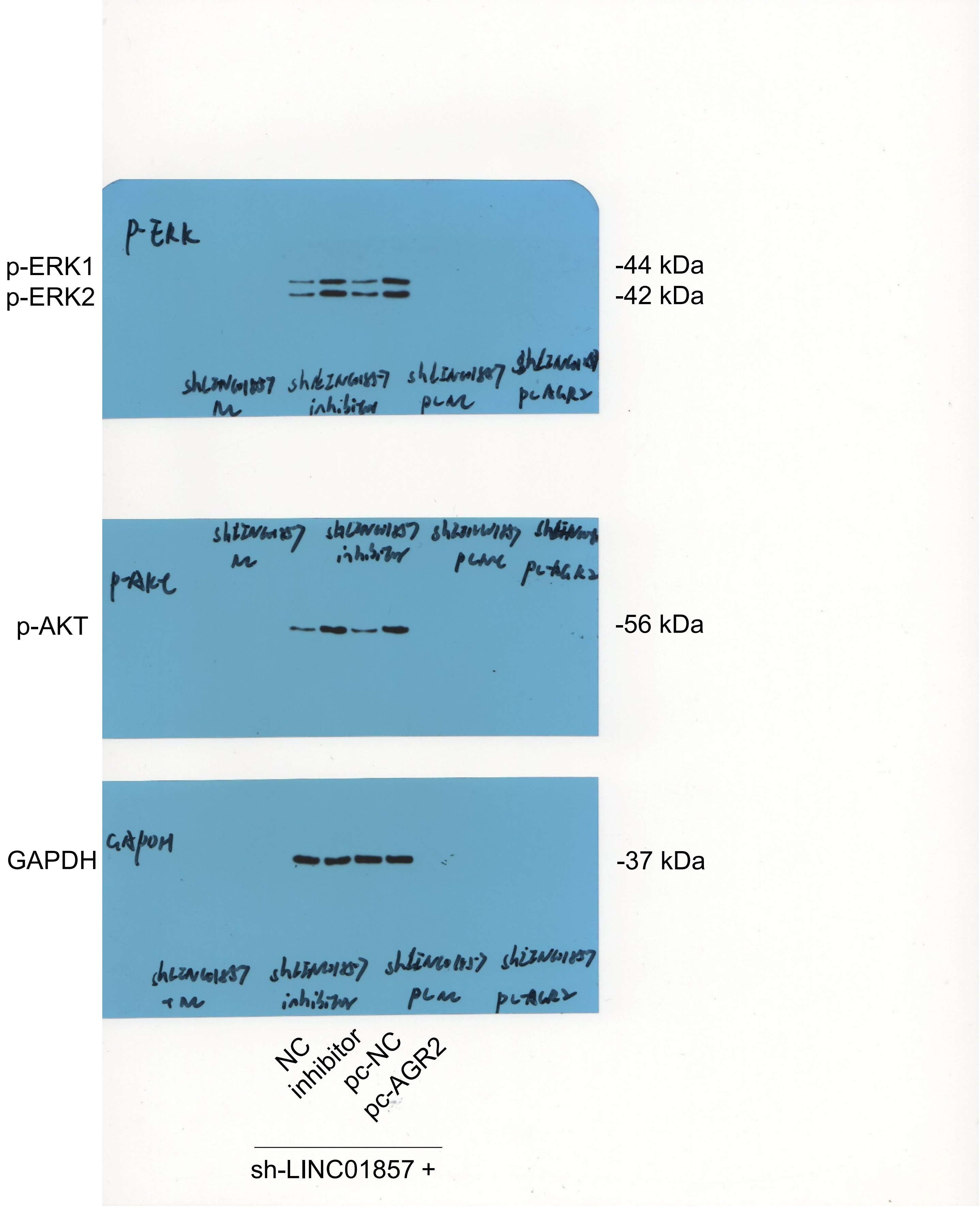

Supplement: S1 Raw images — (PDF) [file pone.0258312.s001.pdf]
