# Supplementary material for: Role of long intergenic non-protein coding RNA 01857 in hepatocellular carcinoma malignancy via the regulation of the microRNA-197-3p/anterior GRadient 2 axis
Source: PLoS One. 2021 Nov 18;16(11):e0258312. doi: 10.1371/journal.pone.0258312 (PMC8601473; doi:10.1371/journal.pone.0258312)
Supplement: Attachment — Submitted filename: Review_RNA 01857 in hepatocellular carcinoma malignancy .docx [file pone.0258312.s002.docx]

Review:

PLoSONE

Role of long intergenic non-protein coding RNA 01857 in hepatocellular carcinoma malignancy via regulating the microRNA-197-3p/anterior GRadient 2 axi.

To Editors

The aim of this study is to understand role of long intergenic non-protein coding RNA (LINC) 01857, weather in loss or gain of function, in hepatocellular carcinoma (HCC) proliferation and apoptosis. According to this study, there are more ways than one in which LINC 0187 may be operating to promote HCC. One of these mechanisms is through protein-protein interaction with (miR)-197-3p and AGR2. Grammatical errors abound everywhere in the manuscript. This study may contribute to our understanding of the different mechanism for HCC development and progression.

To Authors

The study is interesting and useful in deciphering the contributors to HCC development and progression. However, there are several obstacles in achieving clarity in the manuscript preparation including numerous grammatical errors. In the Introduction: “Except for chronic hepatitis B or C virus infection, unbalanced lifestyle in rich regions and the lack of dietary aflatoxin uptake in underdeveloped economies are all boosters for HCC expansion” is not clearly stated. It would be better to state that “…lack of dietary aflatoxin uptake and elimination….”

The sentence: “Furthermore, based on the effect of LINC01857, its downstream network is researched” needs more refining. It seems an incomplete sentence.

In the Methods: “Dual-luciferase reporter gene assay”- the letter “e” appear as “c”s. These substitutions between the letters “c” and “e” occur throughout the manuscript including in the Discussion.

In the Results: The caption: “LINC01857 under-expression inhibits HCC cell proliferation and promotes apoptosis” implies that silencing of LINC0857 is a direct mechanism, which at this point in the experiment is premature and not accurate. It is better stated that” LINC01857 under-expression leads to a blockage in HCC cell proliferation and promotes apoptosis.”

Fig. 1A is not clear. In fact, several the figures are blurry including #a,3B, etc.

Also, the Discussion section needs more alignment with the experimental results obtained instead of regurgitating the contents of the Introduction. The logistical writing of the manuscript will add significantly to the experimental output of the study which is very important.

The evidence for physical protein-protein interaction between LINC01857 and microRNA (miR)-197-3p and between miR-197-3p and anterior GRadient 2 (AGR2) needs more convincing results - perhaps using a pull-down assay since this study seems to show a novel interaction.
