# Supplementary material for: Role of long intergenic non-protein coding RNA 01857 in hepatocellular carcinoma malignancy via the regulation of the microRNA-197-3p/anterior GRadient 2 axis
Source: PLoS One. 2021 Nov 18;16(11):e0258312. doi: 10.1371/journal.pone.0258312 (PMC8601473; doi:10.1371/journal.pone.0258312)
Supplement: Attachment — Submitted filename: Response to reviewers.docx [file pone.0258312.s003.docx]

Dear Editor and Reviewers,

We thank you very much for giving us an opportunity to make the revision, and thank you very much for your comments and opinions. The reviewer’s comments were highly insightful and enabled us to greatly improve the quality of our manuscript. We have revised the manuscript, according to the comments and suggestions of reviewers and editor, and responded point by point to the comments.

With regard to the reviewers’ comments and suggestions, we’d like reply as follows

Reviewers' comments:

Reviewer's Responses to Questions

**Comments to the Author**

1. Is the manuscript technically sound, and do the data support the conclusions?

The manuscript must describe a technically sound piece of scientific research with data that supports the conclusions. Experiments must have been conducted rigorously, with appropriate controls, replication, and sample sizes. The conclusions must be drawn appropriately based on the data presented.

Reviewer #1: Yes

Reviewer #2: Yes

2. Has the statistical analysis been performed appropriately and rigorously?

Reviewer #1: Yes

Reviewer #2: Yes

3. Have the authors made all data underlying the findings in their manuscript fully available?

The [PLOS Data policy](http://www.plosone.org/static/policies.action#sharing) requires authors to make all data underlying the findings described in their manuscript fully available without restriction, with rare exception (please refer to the Data Availability Statement in the manuscript PDF file). The data should be provided as part of the manuscript or its supporting information, or deposited to a public repository. For example, in addition to summary statistics, the data points behind means, medians and variance measures should be available. If there are restrictions on publicly sharing data—e.g. participant privacy or use of data from a third party—those must be specified.

Reviewer #1: Yes

Reviewer #2: Yes

4. Is the manuscript presented in an intelligible fashion and written in standard English?

PLOS ONE does not copyedit accepted manuscripts, so the language in submitted articles must be clear, correct, and unambiguous. Any typographical or grammatical errors should be corrected at revision, so please note any specific errors here.

Reviewer #1: Yes

Reviewer #2: No

5. **Review Comments to the Author**

Please use the space provided to explain your answers to the questions above. You may also include additional comments for the author, including concerns about dual publication, research ethics, or publication ethics. (Please upload your review as an attachment if it exceeds 20,000 characters)

Reviewer #1: The authors have shown that role of long intergenic non-protein coding RNA 01857 in hepatocellular carcinoma via regulating the microRNA-197-3p/AGR2. The work is really interesting, however there are a few minor mistakes which needs to be rectified.

1. Some of the fonts used in the figures are not clear (For eg, Fig 1a, 3a, 3d etc.). I recommend you to provide a better figure for 3c.

**Response:** Thank you for your review. We have modified the fonts and provided higher quality pictures to make the pictures look clearer and easier to understand. Please review again.

2. Please provide the sequences of siRNAs used in the study.

**Response:** Thank you for your suggestion. We have added the sequence to the corresponding method. Please review again.

3. Mention the details of the ELISA kit used in this study.

**Response:** Thanks for your reminding. We have mentioned the details of the ELISA kits.

4. How much total RNA was used for the reverse transcription?

**Response:** Thank you for your question. 2 μg RNA was reverse transcribed into cDNA, which we have described in the method.

5. It is better to write “miRNA”, not “miRs”.

**Response:** Thanks for your comments. We have replaced “miRs” with “miRNA”.
6. Some typos are there in the manuscript.
a. “TRIzol”
b. In some places you have written “tumour” and in some other places “tumor”. Please maintain a consistency.
c. Spelling mistake: “behavirs”

**Response:** Thanks very much for your review and comments. We have revised these typos in the manuscript.

Reviewer #2: The study is interesting and useful in deciphering the contributors to HCC development and progression. However, there are a number of obstacles in achieving clarity in the manuscript preparation including numerous grammatical errors. In the Introduction: “Except for chronic hepatitis B or C virus infection, unbalanced lifestyle in rich regions and the lack of dietary aflatoxin uptake in underdeveloped economies are all boosters for HCC expansion” is not clearly stated. It would be better to state that “…lack of dietary aflatoxin uptake and elimination….”

The sentence: “Furthermore, based on the effect of LINC01857, its downstream network is researched” needs more refining. It seems an incomplete sentence.

**Response:** Thanks. We have refined this sentence. Please review again.

In the Methods: “Dual-luciferase reporter gene assay”- the letter “e” appear as “c”s. These substitutions between the letters “c” and “e” occur throughout the manuscript including in the Discussion.

**Response:** Thanks. We have corrected these errors.

In the Results: The caption: “LINC01857 under-expression inhibits HCC cell proliferation and promotes apoptosis” implies that silencing of LINC0857 is a direct mechanism, which at this point in the experiment is premature and not accurate. It is better stated that” LINC01857 under-expression leads to a blockage in HCC cell proliferation and promotes apoptosis.”

**Response:** Thank you very much. We have revised this caption accordingly.

Fig. 1A is not clear. In fact, a number of the figures are blurry including #a,3B, etc.

**Response:** Thank you for your review. We have checked all the pictures and provided higher quality pictures.

Also, the Discussion section needs more alignment with the experimental results obtained instead of regurgitating the contents of the Introduction. The logistical writing of the manuscript will add significantly to the experimental output of the study which is very important.

**Response:** Thank you for your professional advice. We revised the discussion part to make it more logical. Please review it again.
The evidence for physical protein-protein interaction between LINC01857 and microRNA (miR)-197-3p and between miR-197-3p and anterior GRadient 2 (AGR2) needs more convincing results - perhaps using a pull down assay since this study seems to show a novel interaction.

**Response:** Thank you for your review. According to your suggestion, we supplemented the RNA pull-down experiment to confirm the binding relationship between LINC01857 and miR-197-3p and miR-197-3p and AGR2 (Fig. 3F), and further verified the ceRNA network composed of linc01857/miR-197-3p/AGR2 in hepatocellular carcinoma. Thanks again.

If you have any queries, please do not hesitate to contact us.

Once again, thank you very much for your time spent on this manuscript.

Looking forward to hearing from you.

Your sincerely,

Ping Xu

drxuping244@163.com
